# Mathematical Modeling Predicts How Proteins Affect Cellular Communication

**DOI:** 10.1371/journal.pbio.0000032

**Published:** 2003-10-13

From the moment its life begins, the fate of a multicellular organism depends on how well its cells communicate. Proteins act as molecular switchboard operators to keep the lines of communication open and the flow of cellular messages on track. But charting the protein interactions, signaling pathways, and other elements that regulate these networks is no small feat. Previous efforts have been hampered by the lack of quantitative data—measurements of signal duration, amplitude, and fluctuation—on these regulatory pathways.

Hoping to fill in some of the quantitative gaps, Marc Kirschner of Harvard Medical School, Reinhart Heinrich of Humboldt University Berlin, and colleagues developed a mathematical model as a framework for understanding the quantitative relationships among signaling proteins. To do this, they focused on a well-studied signaling pathway, the Wnt pathway, which plays a role both in various stages of embryonic development and in carcinogenesis. The researchers chose the Wnt pathway in part because a lot is known about it and in part because they could collect enough of the additional measurements they needed to build a solid model from experiments. And like most signaling pathways, Wnt is highly conserved. Consequently, developing tools that elucidate the Wnt pathway will not only provide insights into this important pathway, but have implications for understanding other communication pathways in animals from jellyfish to humans.

To get the additional measurements needed to build their model, the researchers reproduced aspects of the Wnt pathway in the cytoplasm of unfertilized frog eggs. Among the new data collected from these experiments were measurements of the concentrations of scaffold proteins, which bring other components in a pathway together by providing an interaction surface. Strikingly, they found that the principal scaffold proteins involved in the pathway, axin and adenomatous polyposis coli (APC), occur in dramatically different concentrations and perform their jobs in different ways. After a series of refinements based on additional experiments, the model could not only simulate the behavior of the main players in the pathway—both in the absence and presence of a Wnt signal—it also suggested why the two scaffold proteins are present in different concentrations. Axin occurs at very low concentrations relative to the other proteins in the pathway and is likely to bind with them randomly, while APC occurs in similar concentrations and probably binds with the other components in an ordered manner. Because the proteins axin interacts with are also involved in other signaling pathways, the authors propose that the low level of axin here may help the pathways retain their modularity, preventing the Wnt pathway from interfering with the other pathways.

These findings demonstrate that modeling can offer powerful new insights into the workings of complex signaling systems, cutting through the static to pick up important signals even in those pathways that are well understood. The results have important implications for developmental biology and human disease: The Wnt pathway is often activated during carcinogenesis—and mutations in several of these signaling proteins have been linked to colon cancer—suggesting that cancer can develop when signals in the Wnt circuitry somehow get crossed. By predicting how quantitative factors may influence the behavior of signaling networks, mathematical models such as this could shed light on the role that breakdowns in cellular communication play in carcinogenesis. The researchers argue that future attempts to characterize these complex networks must incorporate quantification measurements, and their modeling efforts suggest ways to do that.

**Figure:**
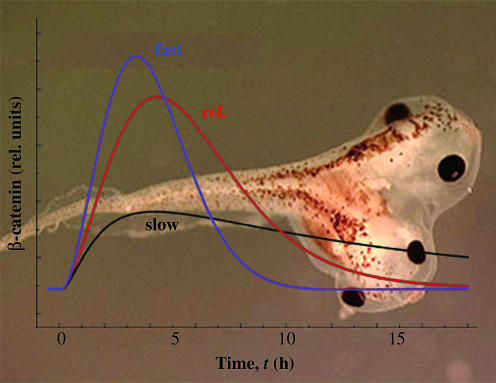
Understanding Wnt signaling through molecular modeling

